# Editorial: Multidimensional benefits of the Mediterranean diet across the lifespan and cultures

**DOI:** 10.3389/fnut.2026.1854446

**Published:** 2026-06-03

**Authors:** Cristina Pederiva, Giuseppe Banderali

**Affiliations:** 1 Pediatrics Unit, Clinical Service for Dyslipidemias, Study and Prevention of Atherosclerosis in Childhood, ASST-Santi Paolo e Carlo, Milan, Italy; 2 Università degli Studi di Milano, Milan, Italy

**Keywords:** culture, health, Mediterranean diet, prevention, quality of life

The Mediterranean diet considers not only dietary habits but also lifestyle factors, such as physical activity, sleep quality, and social interaction, while respecting cultural traditions (1, 2). It is therefore the subject of scientific research with a wide range of applications, particularly in the fields of prevention and public health (3–6).

This *Research Topic* includes 11 scientific papers: six are original research studies, one is a clinical trial, one is a conceptual analysis, and three are review articles. These *Research Topics* highlight the many health benefits of the Mediterranean diet.

The study by Martin-Llaguno et al. examined the relationship between adherence to the Mediterranean diet and body mass index (BMI) in a cohort of adults. The authors confirm the inverse correlation between adherence to the Mediterranean diet, as assessed by the 14-item Mediterranean Diet Adherence Screener (MEDAS) questionnaire, and BMI, as previously reported in the literature. The authors also report that subjects with high BMI have lower dietary quality in terms of fiber, micronutrients, and vitamins compared to normal-weight subjects. They also highlight that these differences remain unchanged even after adjusting for sex, educational level, labor status, physical activity, and energy intake.

Research by Spanish authors Cueto-Galàn et al. supports this view. They investigated the association between the Mediterranean diet and fatty liver disease. The authors evaluated a large group of adult participants from the PREvención con DIeta MEDiterránea (PREDIMED) study who were monitored for at least 5 years. Adherence to the Mediterranean diet was assessed using the MEDAS questionnaire, and steatosis was evaluated using the Hepatic Steatosis Index (HSI). Results showed improved adherence to the Mediterranean diet over the 5-year follow-up period, with improvements in caloric intake, alcohol consumption, and smoking. Blood pressure values improved, and cholesterol levels decreased. Concurrently, the authors documented a significant reduction in hepatic steatosis, which they quantified as a decrease in the HSI per unit increase in adherence to the Mediterranean diet (MedDiet). The authors conclude by emphasizing that the Mediterranean diet is an effective strategy for preventing and treating hepatic steatosis and the associated metabolic abnormalities.

Baroutis et al. conducted a systematic review investigating the potential preventive role of the Mediterranean diet against preeclampsia. Their analysis of five observational studies and seven randomized controlled trials (RCTs) revealed a protective association between the Mediterranean diet and preeclampsia. The main protective effects appear to stem from the diet's distinctive characteristics, such as its anti-inflammatory and antioxidant properties, its ability to modulate angiogenic factors and improve endothelial function, and its well-known ability to improve metabolic parameters. The authors suggest that the Mediterranean diet could be proposed as a promising non-pharmacological treatment for preeclampsia.

Another study (Xia et al.) examined the relationship between adherence to the Mediterranean diet and cancer-related fatigue. This is a cross-sectional analysis of data from the National Health and Nutrition Examination Survey (NHANES) cohort; adherence to the Mediterranean diet was assessed using the Alternative Mediterranean Diet Adherence (AMED) score calculated from a 24-h dietary recall, while fatigue was estimated using the Patient Health Questionnaire Depression Scale (PHQ-9). The authors reported an inverse correlation between the AMED score and cancer-related fatigue, which was confirmed by applying different statistical models (*p* for trend < 0.05). Evaluating specific dietary components revealed an inverse correlation between alcohol consumption and fatigue. A subgroup analysis showed that diabetes, educational level, and cancer type can modulate the relationship between the AMED score and cancer-related fatigue.

A working group of various specialists (Hwalla et al.) evaluated the multiple indices of adherence to the Mediterranean diet. The authors assessed the strengths and limitations of each index, particularly noting the “incompatibility” between them. They argue that this incompatibility stems from the inclusion of non-homogeneous items, making the indices incomparable. The authors proposed and developed a new, unified score called the Unified Mediterranean Diet Score (UMEDS) to eliminate these inconsistencies. The UMEDS considers ten key food groups (whole grains, fruits, vegetables, dairy products, fish, legumes, olive oil, nuts and seeds, poultry, and red meat), assigning a score based on a dose expressed in grams. The UMEDS also considers lifestyle factors, such as physical activity, hours of sleep, and social interaction, as well as the specific cultural context, evaluated through the consumption of foods typical of the individual's cultural background. The authors emphasize that this index is comprehensive but acknowledge limitations, such as lack of validation and evaluation in grams rather than portions, which could limit its application in clinical practice. The authors conclude by expressing hope that the proposed score will improve the assessment of adherence to the Mediterranean diet in the future.

On an international scale, Boujelbane et al. conducted a survey in 10 Mediterranean countries to assess adherence to the Mediterranean diet using the MedLife Index, a validated tool that assesses adherence to Mediterranean dietary patterns and lifestyle behaviors through three categories: Mediterranean food consumption, MedDiet habits, and lifestyle behaviors. The analysis integrated validated tools for evaluating physical activity, sleep quality, mental health, life satisfaction, social participation, and technology use. Potential gender differences were the focal point of the analysis of the results. Overall, the data did not show differences in adherence between men and women as assessed by the total MedLife score. However, women demonstrated better adherence to consuming specific dietary components, while men showed greater physical activity and social participation. The authors conclude by emphasizing the need for a differentiated public health approach that considers the behavioral and psychosocial differences between women and men when adopting the Mediterranean model.

In conclusion, this *Research Topic* has examined various aspects of the Mediterranean diet. Some works have confirmed a correlation between the Mediterranean diet, body mass index (BMI), and fatty liver disease. These studies highlight the diet's protective role in important clinical and public health aspects. Other studies have identified the potential positive impact of the Mediterranean diet on specific health conditions, including pregnancy and chronic diseases such as cancer. Finally, some authors have delved deeper into evaluating the indices of adherence to the Mediterranean model in greater detail. They have examined the model as a cultural model and the differences in adherence between men and women. Taken together, these studies have significantly advanced our scientific understanding of this ancient dietary and cultural model, which remains highly relevant today.
